# Application of Inertial Measurement Units and Machine Learning Classification in Cerebral Palsy: Randomized Controlled Trial

**DOI:** 10.2196/29769

**Published:** 2021-10-20

**Authors:** Siavash Khaksar, Huizhu Pan, Bita Borazjani, Iain Murray, Himanshu Agrawal, Wanquan Liu, Catherine Elliott, Christine Imms, Amity Campbell, Corrin Walmsley

**Affiliations:** 1 School of Electrical Engineering, Computing and Mathematical Sciences Curtin University Bentley Australia; 2 School of Intelligent Systems Engineering Sun Yat-sen University Shenzhen China; 3 School of Allied Health Curtin University Bentley Australia; 4 The University of Melbourne Melbourne Australia

**Keywords:** inertial measurement unit, wearable sensors, biomedical sensors, machine learning, human joint measurement, occupational therapy, range of motion, wearable, sensor, children, cerebral palsy, therapy, disability

## Abstract

**Background:**

Cerebral palsy (CP) is a physical disability that affects movement and posture. Approximately 17 million people worldwide and 34,000 people in Australia are living with CP. In clinical and kinematic research, goniometers and inclinometers are the most commonly used clinical tools to measure joint angles and positions in children with CP.

**Objective:**

This paper presents collaborative research between the School of Electrical Engineering, Computing and Mathematical Sciences at Curtin University and a team of clinicians in a multicenter randomized controlled trial involving children with CP. This study aims to develop a digital solution for mass data collection using inertial measurement units (IMUs) and the application of machine learning (ML) to classify the movement features associated with CP to determine the effectiveness of therapy. The results were calculated without the need to measure Euler, quaternion, and joint measurement calculation, reducing the time required to classify the data.

**Methods:**

Custom IMUs were developed to record the usual wrist movements of participants in 2 age groups. The first age group consisted of participants approaching 3 years of age, and the second age group consisted of participants approaching 15 years of age. Both groups consisted of participants with and without CP. The IMU data were used to calculate the joint angle of the wrist movement and determine the range of motion. A total of 9 different ML algorithms were used to classify the movement features associated with CP. This classification can also confirm if the current treatment (in this case, the use of wrist extension) is effective.

**Results:**

Upon completion of the project, the wrist joint angle was successfully calculated and validated against Vicon motion capture. In addition, the CP movement was classified as a feature using ML on raw IMU data. The Random Forrest algorithm achieved the highest accuracy of 87.75% for the age range approaching 15 years, and C4.5 decision tree achieved the highest accuracy of 89.39% for the age range approaching 3 years.

**Conclusions:**

Anecdotal feedback from Minimising Impairment Trial researchers was positive about the potential for IMUs to contribute accurate data about active range of motion, especially in children, for whom goniometric methods are challenging. There may also be potential to use IMUs for continued monitoring of hand movements throughout the day.

**Trial Registration:**

Australian New Zealand Clinical Trials Registry (ANZCTR) ACTRN12614001276640, https://www.anzctr.org.au/Trial/Registration/TrialReview.aspx?id=367398; ANZCTR ACTRN12614001275651, https://www.anzctr.org.au/Trial/Registration/TrialReview.aspx?id=367422

## Introduction

### Background

Cerebral palsy (CP) is a condition that affects a person’s ability to move [1,2]. It occurs as a result of injury to the developing brain during pregnancy or a short time after birth [3]. CP presents with different characteristics in different people, as the damage to the brain is not identical in every person‎ [1]. The movement difficulties experienced by people with CP are divided into three main categories: spastic motor type, in which muscles appear stiff and tight (most common); dyskinetic type, which involves involuntary movement patterns; and ataxic type, which involves uncoordinated muscle movements that can affect balance and sense of positioning in space ‎[3,4]. The level of severity and combination of symptoms can differ from person to person [5]. For example, one person could have weakness in one hand, which can lead to difficulty in writing or tying shoelaces, whereas another person may have little control over their movement or speech because CP can also affect the person’s ability to coordinate the muscles around the mouth and tongue [5].

There are many different clinical classification systems for upper limb function in children with CP with different levels of complexity. In a review by McConnell et al [6], 18 different clinical classification systems were identified and reviewed according to whether they classified function or deformity and by considering the quality of psychometric evidence for each method. These methods were rated based on the clinical utility of each system using previously published tools [6]. An example of clinical classification system is House [7] classification, which contains four categories of thumb deformities. Another example of clinical classification is that by Green and Banks [8], which contains four subgroups of poor, fair, good, and excellent based on the use of the hand by the individual with CP. These classification methods demonstrate the complexity of clinical classification of hand movement in children with CP and the diverse approaches taken to achieve it.

As of early 2021, there is no single method for completely curing or preventing CP. Public health measures such as mandatory seatbelts, pool fencing, and rubella vaccinations are among the prevention methods currently in use [9]. Physiotherapy and occupational therapy focus on encouraging a person’s day-to-day movement skills and abilities, such as sitting, walking, dressing, and toileting, and use a range of specialist interventions such as movement and goal-directed training and provision of equipments, such as walking frames, wheelchairs, supportive seating, footwear, and orthotics [9]. When studying children with CP, range of motion, which is the capability of a joint to go through its complete spectrum of movement, may become a crucial component of research. Passive range of motion can be defined as the range of motion when an external force causes movement of the joint and is the maximum range of motion, whereas active range can be achieved when opposing muscles contract and relax, resulting in child- or person-initiated joint movement [10].

Occupational therapists use upper limb orthoses for children with CP who have muscle overactivity caused by spasticity, but there is little evidence of the long-term effects of these methods [11]. The clinical rationale is that the orthoses help preserve the range of movement; however, they are complex to construct, expensive, and can cause discomfort for the children wearing them [11]. To address the need for robust evidence, a multicenter randomized controlled trial (RCT) is being used to evaluate the effectiveness of wrist hand orthoses to prevent loss of range of movement in children with CP (see *Experiment Setup and Data Collection* for details). This RCT used inertial measurement units (IMUs) to measure active movement in children with CP, to address two measurement problems: (1) the complex movement patterns of children with CP make it difficult for therapists to accurately apply typical clinical measures, such as a goniometer (an instrument that measures the available range of motion at a joint) and (2) young children’s small hands and difficulty following detailed movement instructions make it difficult to achieve reliable measurements.

### Existing Methods

General movement assessment is used, which is a noninvasive and cost-effective method for identifying babies at risk of CP [12]. This assessment is done by recording a 3- to 5-minute video of an awake infant lying on their back while they were calm and alert without the presence of toys and pacifiers. Parents can be present and record the video, but they should not interact with their babies. This video is then observed and assessed by trained health professionals to detect signs of the disorder [3,12]. This process becomes easier when infants grow older, as they can follow the instructions of the medical professionals to perform different tasks so that their movement can be monitored. This assessment is mainly used as a diagnostic tool for the early detection of CP, and it is not used to quantify the range of movement or motion.

In clinical research, the goniometer and inclinometer are used to measure joint angles in children with CP [13]. A goniometer is an instrument that measures the joint angle, and depending on the nature of the experiment, it can measure the available range of motion at a joint. It can be used to monitor changes in joint angles in clinical settings [14]. The traditional method of using angle-measuring tools is not accurate and reliable, according to some recent studies [13]. Accurately measuring range of motion (ROM) is an important part of clinical assessment as this information is used to guide treatment plans, determine treatment efficacy, and monitor individual’s response to treatment [15]. Goniometric measures rely on the ability of the clinician to accurately palpate bony landmarks and visually estimate the alignment of the axis and arms of the goniometer to the joint that is being measured. Goniometers are versatile, reliable, and widely used, irrespective of their measurement errors of up to 15 degrees. However, for active movement, the use of goniometers is very difficult, and their use may not be possible in populations that are unable to respond to instructions reliably [15].

A general approach for capturing movement is the use of digital technologies, such as motion capture. Motion capture (also referred to as mo-cap or mocap) is the process of digitally recording the movement of people [16]. It is used in entertainment, sports, medical applications, ergonomics, and robotics. In filmmaking and game development, it refers to the recording actions of actors for animations or visual effects. It is also referred to as performance capture when it includes a full body, face, and fingers or captures subtle expressions [16]. The equipment required for motion capture is extremely costly and is not commonly available in a typical hospital; for example, according to Thewlis et al [17], a simple Vicon system [18] cost approximately Aus $250,000 (US $268,605.52) in 2011 [17]. Even if the equipment is available, it may be difficult to take children to these motion analysis laboratories to conduct measurements. Another limitation is the need for expert staff to run the laboratories for the motion analysis of hand movement.

Another approach is to measure gesture control using electronic sensors, such as infrared (IR) light-emitting diodes. Gesture recognition software for advanced smartphones was presented in the paper found in the study by Kong et al [19]. The leap motion sensor uses IR sensors to scan finger movements with a typical field of view of 140°×120° [20]. This method is mostly applied in the entertainment industry, so it does not meet the need for accuracy in capturing the movement of people with CP.

With the development of inertial sensor technologies, IMU-based motion capture systems have been introduced in the study of human motion. IMUs comprise an accelerometer, gyroscope, and magnetometer that are connected to a microcontroller and can be used to capture orientation. In recent years, there have been several IMU-based motion capture research studies, such as studies of gait modulation in patients with foot drop problems [21] and human activity recognition using thigh angle derived from a single thigh-mounted IMU data [2]. The use of IMUs for hand movement in free space is currently underdeveloped, primarily due to the lack of a clear calibration reset point compared with gait analysis. Another benefit of IMU solutions is flexibility in the collection window. From a practical point of view, the data measured during any session using motion capture technologies or any nonportable devices that require the patient to be at a certain location at a certain time, which may not be a period when certain movement characteristics are present or typical. For example, the patient could be *having a good day* or fatigued coincidentally during the clinic visit. IMU measurements outside the predefined time may avoid errors in the data collection. In addition, patient’s compliance would potentially increase in the case of children, where their movement is taking place in their home environment compared with organized clinic visits. The challenge would then be to filter a larger data set to remove outliers, which is already a problem even when clinicians are involved. Therefore, the IMU data collection needs to be streamlined so that data can be captured easily without any need for clinical or technical expertise.

An overview of all the relevant existing methods, including their advantages and disadvantages can be seen in Table 1.

**Table 1:** Evaluation of existing methods.

Type of approach	Advantages	Disadvantages
Goniometers [14]	Low cost Can provide measurements very quickly	Lack of accuracy Does not provide long-term tracking of movement unless repeated multiple times Difficult when children are involved
Video capture [16]	Very accurate Can provide real-time orientation and active movement	Very costly Continued monitoring is not possible outside the motion capture studio Long set up time Facilities are not available to everyone
IR^a^ LED^b^ gesture recognition [20]	Low cost Portable	Lack of accuracy Not possible for continued monitoring Mostly developed for entertainment use
IMU^c^ [22]	Low cost Can provide a reasonably accurate orientation frame Low power consumption Portable	IMUs drift over time The postprocessing of IMU data can be lengthy

^a^IR: infrared radio.

^b^LED: light-emitting diode.

^c^IMU: inertial measurement unit.

### Contribution of the Paper

This paper presents collaborative research between the Department of Electrical Engineering and Computing at Curtin University and the investigator team of a multicenter RCT involving children with CP [11]. The novelty of this work is the mass data collection and application area of the sensor system. To achieve this goal, 2 small, low-cost, custom-built IMUs were developed to capture the hand movements of participants in 2 age groups. The first age group had participants approaching 3 years, and the second age group had participants approaching 15 years. Both groups comprised participants with and without CP. Custom sensors were needed because commercial sensors are costly and do not provide raw sensor data. This means that validation cannot be performed easily. In addition, the use of custom sensors will avoid preprocessing by a third party. The designed sensors were capable of measuring wrist joint movement as the angle difference between 2 parallel sensors, which simplifies a 3D system problem to a 2D one. Therefore, only the relative motion was used, and the impact of the environment was ignored. This approach facilitates a reliable and valid method to capture changes over time. Capturing ROM over time is important because children with CP have secondary musculoskeletal complications, which means they are at risk of losing movement range. The proposed low-cost sensor system could also provide the means for active and continuous tracking of wrist joint movement during usual or predetermined tasks and actions that are currently not possible using traditional goniometric methods.

A second contribution of this paper is the application of ML to raw IMU data to classify the movement features associated with CP without the need to measure Euler, quaternion, and joint measurement calculations. This means that the processing time will be reduced because of using raw data for classification. This classification aims to investigate the existence of characteristics of CP movement, which is different from the clinical classification used for CP as a condition. This classification can also confirm if treatment (in this case, the use of wrist extension) is effective. After the initial data collection, 9 different ML algorithms were used to classify CP as a feature: the Random Forrest algorithm achieved the highest accuracy of 87.75% for the age range approaching 15 years, and C4.5 decision tree achieved the highest accuracy with 89.39% for the age range approaching 3 years. The result of this classification aligns with existing research work in which ML is applied to classify footdrop using IMUs [23]. The results of this project showed that decision tree-based ML algorithms were the most accurate compared with other methods, which could be used as a guideline for similar human joint measurements.

## Methods

### Sensor Development

**Figure 1:**
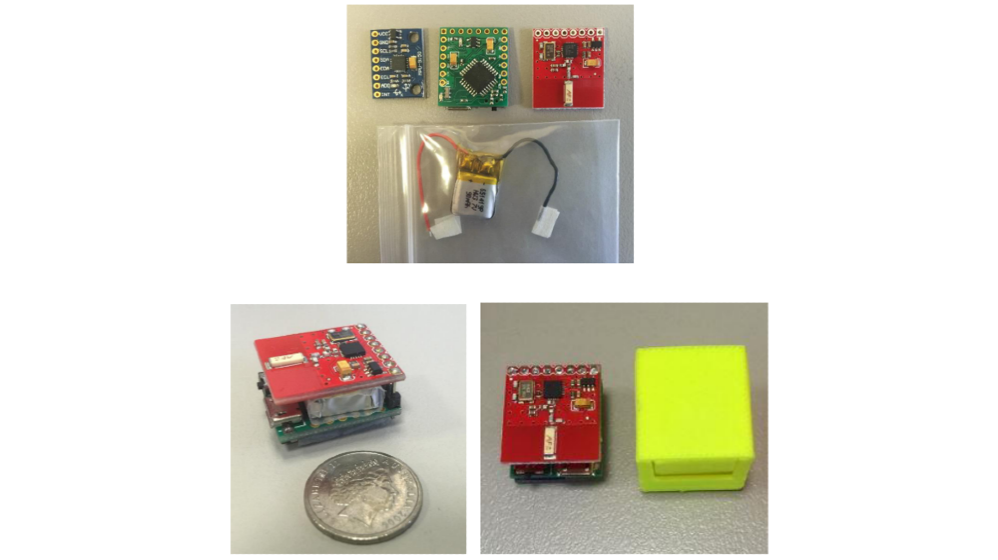
The MPU9150 (blue printed circuit board [PCB]), custom-built Arduino Pro Mini (green PCB), and RF Module (red PCB); a comparison of the inertial measurement unit with an Australian five-cent coin; and the 3D printed case for the sensor [24].

**Figure 2:**
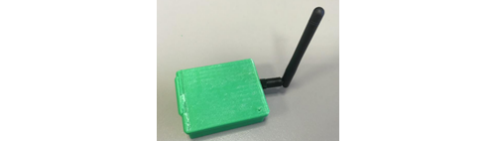
The receiver dongle in the 3D printed case [24].

A custom-built IMU was developed to capture the hand movements of children with CP for this project. The IMU consisted of an MPU 9250, a custom-built Arduino Pro Mini, and a 2.4-GHz radio frequency (RF) radio. Each sensor was powered by a small 90 mAh, 3.7-V rechargeable lithium battery and could support up to 3 hours of nonstop measurement. The custom Arduino Pro Mini was previously designed by Dr Weiyang Xu as part of his thesis titled *Design and Validation of a Portable Wireless Data Acquisition System for Measuring Human Joint Angles in Medical Applications* [24]. The IMU data were captured using a simple receiver dongle that used an RF radio transceiver connected to an Arduino Uno and was read from the serial communication link. Both RF modules were connected using a serial peripheral interface (SPI), and the IMU was connected using an interintegrated circuit (I^2^C) connection. The designed IMU is shown in Figures 1 and 2. A summary of the specifications of the IMU is presented in Table 2. These sensors were validated against a goniometer and Vicon motion capture system, the results of which can be found in the studies by Walmsley et al [15] and Xu et al [25].

**Table 2:** Specification of the inertial measurement unit (IMU).

Electronic Module	Parameter	Value
MPU 9250 IMU	Accelerometer FS range Gyroscope FS range Magnetometer FS range	Range of ±2 g, ±4 g, ±8 g and ±16 g Range of ±250, ±500, ±1000 and ±2000°/sec Range of ±1200 µT
nRF24L01 Transceiver	ISM^a^ band operation Air data rate Programmable output power	2.4GHz 250 kbps, 1 and 2 Mbps 0, −6, −12 or −18 dBm
Arduino Pro mini	Circuit operating voltage Clock Speed Flash memory	3.3 V or 5 V 8 MHz (3.3 V version) or 16 MHz (5 V version) 32 KB
Arduino Uno	Circuit operating voltage Clock Speed Flash memory	5 V 16 MHz 32 KB

^a^ISM: Industrial, Scientific, and Medical.

**Figure 3:**
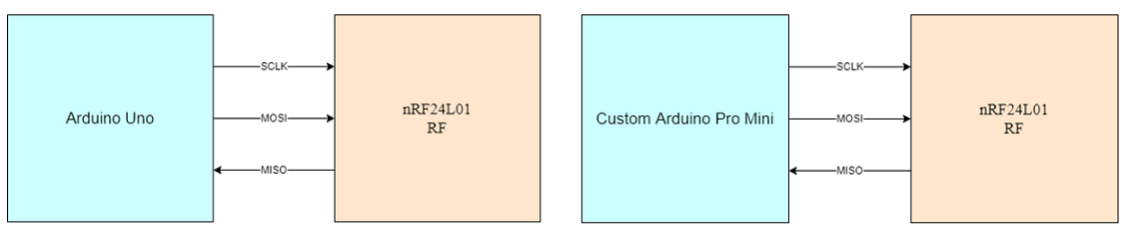
The left diagram shows the serial peripheral interface (SPI) connection between Arduino Uno and the RF module, and the right diagram shows the SPI connection between the custom Arduino Pro mini and the RF module. MISO: Master In Slave Out; MOSI: Master Out Slave In; RF: radio-frequency; SCLK: Serial Clock.

The SPI is a synchronous, full-duplex serial bus standard that was introduced by Motorola to support communication between a master processor and multiple slaves [26]. This protocol used Serial Clock sent by the master to synchronize master and slave; Serial Data Out to stream from the device; Serial Date In to stream into the device; Slave Select to enable slave, which is omitted in point-to-point connotations [26]. The master–slave connection for the RF module is shown in Figure 3. SPI was used to connect the RF module to the custom build module, where Arduino was the master and the RF module was the slave. The same connection was used on the receiver to connect the RF module and Arduino Uno with the Arduino acting as the master and the RF module acting as the slave. This decision was made because of the inclusion of Master In Slave Out and Master Out Slave In data lines that facilitate full-duplex communication, a fast communication speed that can go to 10 Mbps or more; inclusion of push-pull drivers that provide good signal integrity, not limited to 8-bit words for bits transferred; use of master’s clock by the slave, which removed the need for precision oscillators, and lower power requirements compared with other serial buses because of less circuitry.

The designed sensors needed to wirelessly transfer data to avoid hindering the hand movements of the participants in the project. Popular wireless communication technologies include Bluetooth, RF, WiFi, and infrared. The popular frequency range for wireless communication includes subGHz below 1 GHz (for long-range) and 2.4 GHz (for short-range). The proposed joint movement calculation system uses an nRF24L01 RF transceiver [27] (transmitter-receiver integrated on the same chip) module, which operates on a 2.4-GHz frequency band using 125 channels in the frequency range of 2.4 GHz-2.525 GHz. The module uses a license-free industrial, scientific, and medical frequency and can cover a distance of up to 1000 m. To improve the data loss at this crowded frequency band around 2.4 GHz, the nRF24L01 RF transceiver module uses a low noise amplifier [27]. The data rate requirement of the proposed joint movement calculation is not very high. This RF transceiver module is an improvement as it supports data rates in the range of 250 kbps-2 Mbps. The RF transceiver module connects with the Arduino module using SPI through Serial Clock, Master In Slave Out, and Master Out Slave In pins. The nRF24L01 RF transceiver is an ultralow power drawing of 26 µA of current in standby mode and 900 nA of current under down mode [27].

**Figure 4:**
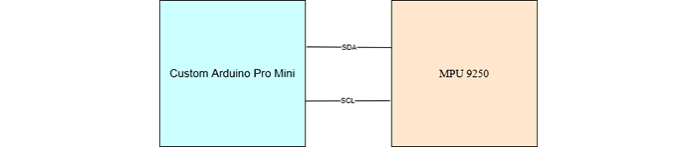
Schematic of the I2C connection between the custom Arduino Pro Mini and the inertial measurement unit. SCL: Serial Clock Line; SDA: Serial Data Line.

**Figure 5:**
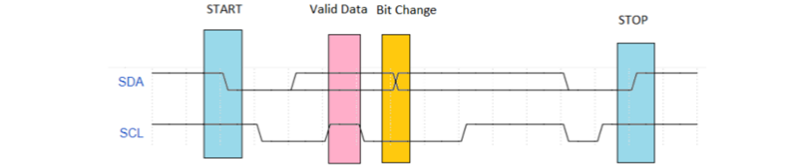
I2C timing diagram. SCL: Serial Clock Line; SDA: Serial Data Line.

The I^2^C bus is a synchronous serial protocol originally developed by Philips Semiconductor (now known as NXP semiconductors) in the early 1980s [26]. The main aim of I^2^C was originally to support the board-level interconnection of ID modules and peripherals [26]. This protocol used serial data, and Serial Clock and ground for a half-duplex connection, which is capable of handling multiple masters and slaves. Serial Clock synchronizes all bus transfers, and serial data carries the data being transferred [26]. The connection of the MPU 9250 module is shown in Figure 4. The structure of the timing diagram for I^2^C is shown in Figure 5. The I^2^C was used to connect the IMU module to the custom-built IMU, with the Arduino acting as the master and the IMU acting as the slave. This decision was made because of the incorporation of Acknowledgment and No Acknowledgment functionality that improves error handling, flexible data transmission rates, addressability of each devices bus, and requiring only 2 signal lines.

The IMUs comprise an accelerometer, gyroscope, and magnetometer. Using sensor fusion techniques, an object’s orientation can be captured using differential equations describing its dynamic behavior, which can be derived from the Newton-Euler by means of the Euler angle parametrization [28]. Quaternion is another method for capturing the orientation of an object, which is a four-element vector that can be used to encode any rotation in a 3D coordinate system [28]. In this study, to simplify calculations, raw acceleration and angular velocity were captured and used to measure the wrist joint angle. The requirements and specifications of this research lead to the selection of IMUs owing to their low cost, low power consumption, and ability to provide orientation with the relevant update rate.

### Joint Angle Calculations

**Figure 6:**
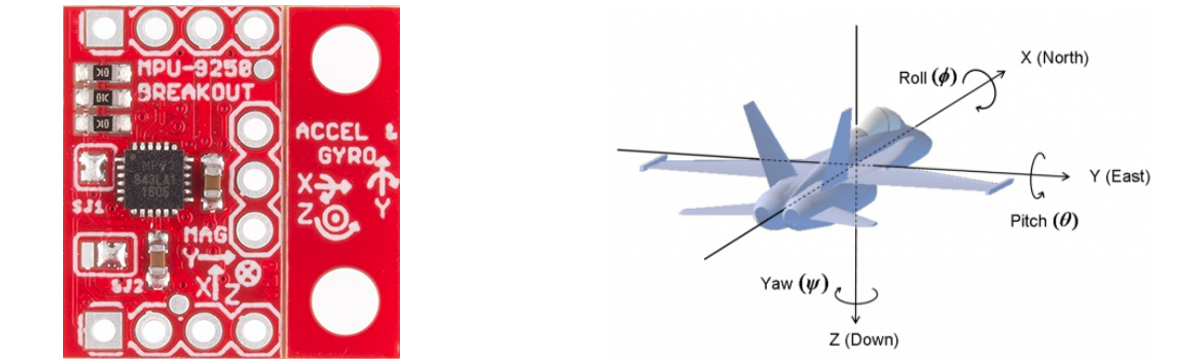
Orientation of the MPU9250 inertial measurement unit chip, where X is Roll, Y is Pitch, and Z is Yaw [29].

**Figure 7:**
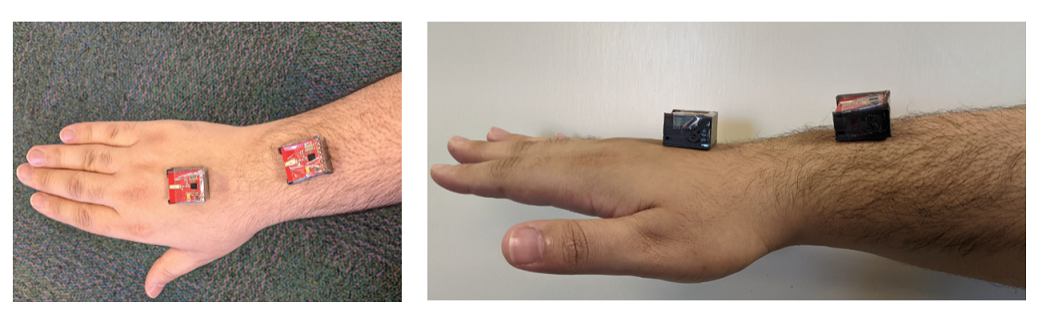
Sensor placement showing sensor 1 connected to the back of the hand and sensor 2 connected above the wrist.

The sensors collected raw acceleration and angular velocity in the X-, Y-, and Z-axes, and the results were postprocessed in MATLAB using a 2-sensor-based joint orientation algorithm. This algorithm shows the difference in relative movements between 2 sensors when they share the same frame and zero position [24]. The Z- and Y-axes of both sensors need to be parallel to each other, so the X-axis of both sensors merge into the wrist center. This means that the wrist joint movement can be measured as the angle difference between the 2 sensors. The use of 2 parallel sensors for joint calculation simplifies the 3D system problem to a 2D one. The orientation of the MPU9250 is shown in Figure 6 [29] and the placement of the sensors is shown in Figure 7.

Using 2 sensors creates a relative system, so the rotation on the Y-axis or the orientation on the X-Z plane can simply be calculated using the following formula:




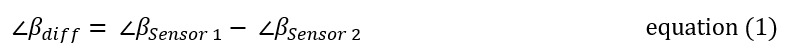




According to the tangent function, the angle of ß can be initially calculated using the acceleration from the X-and Z-axes, where x is the angle between the net acceleration and the acceleration on the X-Z plane. Therefore, the tangent of ß can be calculated as follows:




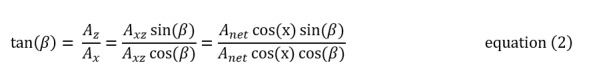




**Figure 8:**
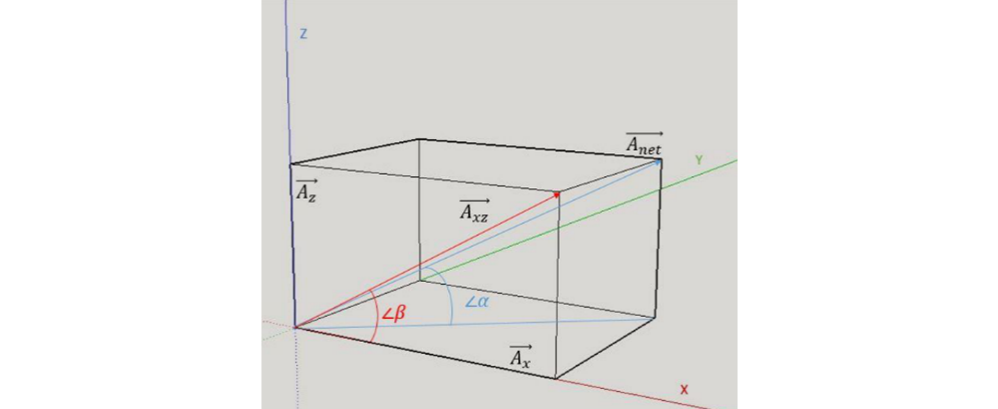
3D system for acceleration.

The angles used in equation (2) can be seen in Figure 8.

The data sample rate for both sensors was set to 100 Hz, which reduced the difference in angular velocity measurements between each sample.

Unlike traditional yaw, pitch, and roll orientation systems, a reference plane was unnecessary in the present algorithm as both sensor axes were aligned so that the joint movement was equivalent to the orientation difference between the sensors. Therefore, only relative motion was used, and the impact from the environment was ignored [24].

The orientation of each individual sensor was calculated using the orientation reading and angle movement during each sampling period, and a complementary filter introduced a high-pass filter to the main orientation tracker and adjusted with a low passed value from the accelerometer’s orientation measurement [24].

As the desired accuracy cannot be achieved by using only the acceleration, sensor fusion was used to increase the measurement accuracy by combining the data from both the accelerometer and the gyroscope. The accelerometer output was independent of each sample during the measurement period; therefore, *θ_zx_*, *θ_yz_*, and *θ_zy_*, which are the projected orientation angles on the X-Z, Y-Z, and Z-Y planes, respectively, were used as rough measurements. The gyroscope’s angular velocity *ω_gf_* was added to describe the actual change between samples and can be calculated after subtracting the average static drift and using a Savitzky-Golay filter to calibrate the moving average drift [24]. The gyroscope’s angular velocity can be calculated using the following formula:




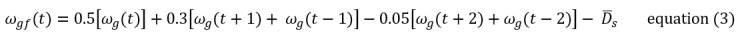




Here, 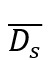 is the average static drift, which can be calculated using the following equation:




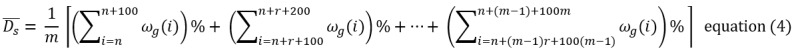




In the formula given above, n, m, and r are random integers and m is larger than 3. The total number of samples needs to be larger than n + (m−1) r + 100 m. These calculations lead to the following sensor fusion algorithm, which is based on a complementary filter:




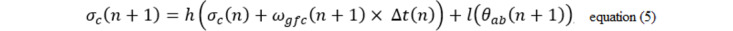




where a, b, and c are the names of thåe measurement axes and n+1 is the current order of the sample. *σ_c_ (n + 1)* is the filtered angle along the c-axis. Therefore, *ω_gfc_* represents the rotation on the c-axis, and *θ_ab_* is the current angle on the a-b plane, which is based on accelerometer measurements. Finally, the combination of high pas factor h and low pas factor l is 1 [24].

The results of these joint calculations were validated in the study by Sharif Bidabadi et al [30] against a 3D Vicon video capture setup. The accuracy of the setup was written in a different paper found in the study by Walmsley et al [15], where a custom-made robotic device with predetermined angles was designed, where the sensors detected peak angles with mean errors ranging from −0.95° to 0.11° when one wearable sensor was static and the other dynamic. When 2 wearable sensors were moving, movement at a higher speed (90°/s) had a mean error range of −2.63° to 0.54° and movement at a slower speed (30°/s) had a mean error range of −0.92° to 2.90° [15].

### Data Preprocessing

The IMU sensors generated time-series data from the accelerometer, gyroscope, and magnetometer around the 3 axes. First, small sections were removed from readings taken at the beginning of the experiments when the IMU sensors were not stabilized. Then, the remaining data collected by each sensor from each experiment in 3 orientations (ie, pitch, row, and yaw) were converted into frequency-domain representations by performing fast Fourier transform. Converting data to the frequency domain can successfully capture the characteristics of gait motion, as shown by similar experiments in [23,31,32], the interval between adjacent readings was approximated as 0.1 seconds and the fundamental frequency was calculated as 1/t_total_, where t_total_ is the total time of the experiment. The amplitude *A*, phase shift *P*, and peak frequency *F* of the first 5 harmonics were collected into a feature vector. The feature vector for each experiment was 1×270, and the 270 features were as follows:




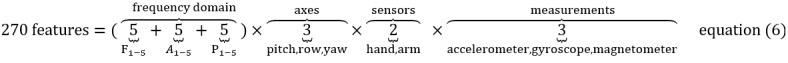




Each experiment was then labeled 0 for a typically developing child and 1 for a child with CP.

### Classification by ML Algorithms

The problem of distinguishing typical hand movements from hand movements of children with CP constitutes a binary classification problem, that is, classification between two classes. Various algorithms can be constructed using different ML methods based on existing data that can be used to classify unseen data. This process is called *training*. Some classical ML algorithms commonly used in engineering problems include linear classifiers such as Naïve Bayes and logistic regression, decision trees such as the C4.5 decision tree and random forest, support vector machine, k-nearest neighbors, and neural networks such as multilayer perceptron and convolutional neural networks. More sophisticated deep neural networks can also be designed for classification problems; however, the size of training data sets is a major concern. Other problems include data bias, overfitting, a lack of computational resources, etc.

To decide between the 2 classes, ML algorithms for binary classification establish decision boundaries that separate the data points in the training data set from the 2 classes. This process relies on optimizing a cost function that varies between the algorithms. Most algorithms, such as logistic regression, support vector machine, decision trees, and neural networks, aim to construct a model with parameters that are learned from the training data set, whereas some algorithms operate directly on the data set, for example, k-nearest neighbors. Although there are numerous libraries and tools offering implementations of ML algorithms [33,34], the performance of the individual algorithm depends on the nature of the problem and the properties of the data set. Choosing the algorithm that performs best for a particular problem is subject to investigation.

### Experiment Setup and Data Collection

As a part of an Australia-wide CP research study called the *Minimising Impairment Trial* (MIT) and *Infant Wrist Hand Orthosis Trial* (iWHOTs), the IMU sensors were used to capture the wrist movements of 2 groups of participants. The MIT trial included children with and without CP aged 5-15 years, and the iWHOT included children aged 6 months to 3 years. These studies were multisite RCTs that aimed to evaluate whether long-term use of rigid wrist or hand orthoses in children with CP, combined with usual multidisciplinary care, could prevent or reduce musculoskeletal impairments, including muscle stiffness or tone and loss of movement range, compared with usual multidisciplinary care alone [11]. IMUs were used as an outcome measure to capture the active wrist ROM. During each assessment session, the participants completed several wrist movement activities such as making a stop sign motion, picking up small objects, playing with toys, pressing a big button, and so on. The aim of these activities was to assess the ROM used during active movement and task performance while data were collected via sensors. In addition, goniometric measurements of the joint movement was collected. The detailed protocol of this research has been published [11] if the reader is interested in more information about the clinical aspects of this trial.

For this project, the aim was to capture CP movement as a feature by ML on the raw IMU data by focusing on the data collected during the stop sign task in the MIT and iWHOT. Each participant was asked to perform a simple stop sign motion to capture the maximum wrist joint angle as well as the maximum range of movement. To achieve this study’s aim, two separate experiments were run using participants who were approaching the age of 3 years from iWHOT and participants who were approaching the age of 15 years from MIT. From MIT, 263 samples from 89 participants with CP and 199 samples of typical movement data captured from 30 participants without CP were used. The participants without CP simulated typical movements to reach 199 samples. From iWHOT, 171 samples from 51 participants with CP and 149 samples from 20 participants without CP were used.

Cross-validation, which is 90% training and 10% testing, were used 10 times to train and test the classifier, which can be seen in the next section of this paper. The CP data were collected by the research teams working on the MIT and iWHOT trial according to ethically approved procedures (HREC REF 201406EP) and with signed, informed consent from all the participants’ parents or guardians. Deidentified data were used to produce ML results, which are analyzed in the *Discussion* section of this paper.

## Results

**Figure 9:**
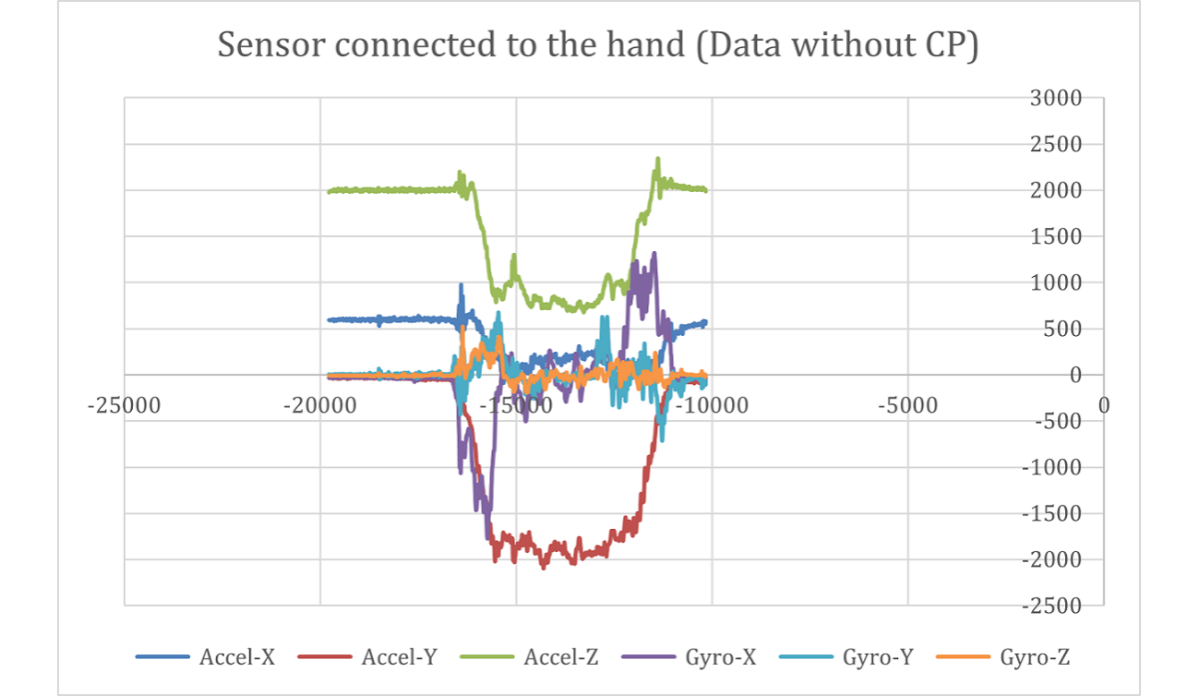
Raw data captured with the sensor connected to the hand (data without CP). CP: cerebral palsy.

**Figure 10:**
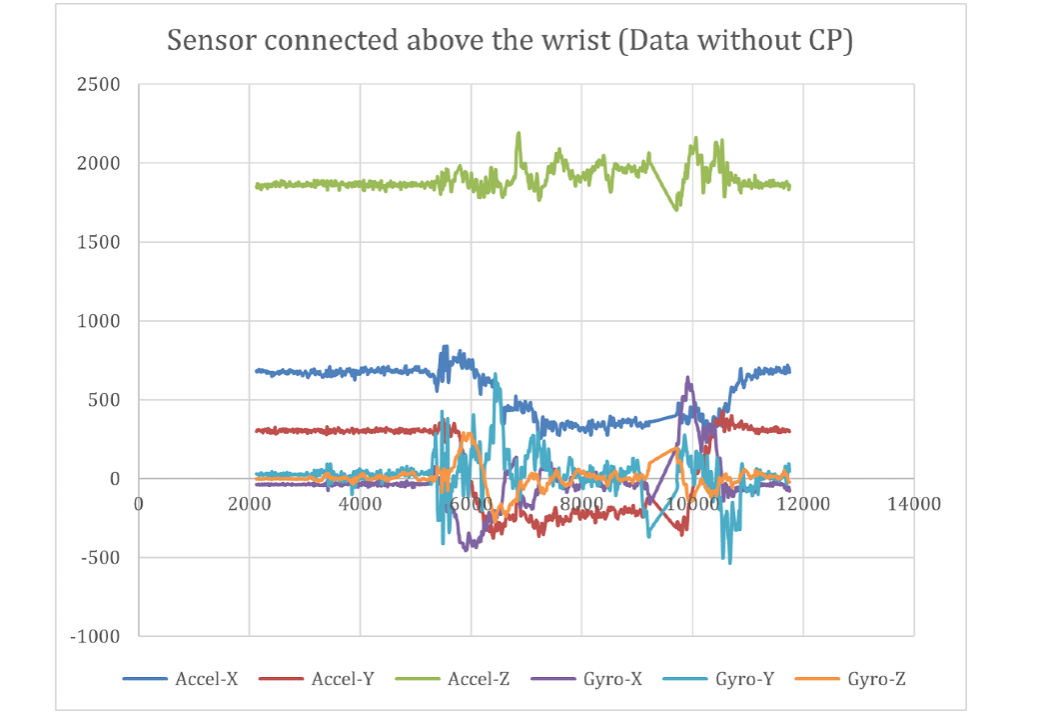
Raw data captured with the sensor connected above the wrist (data without CP). CP: cerebral palsy.

**Figure 11:**
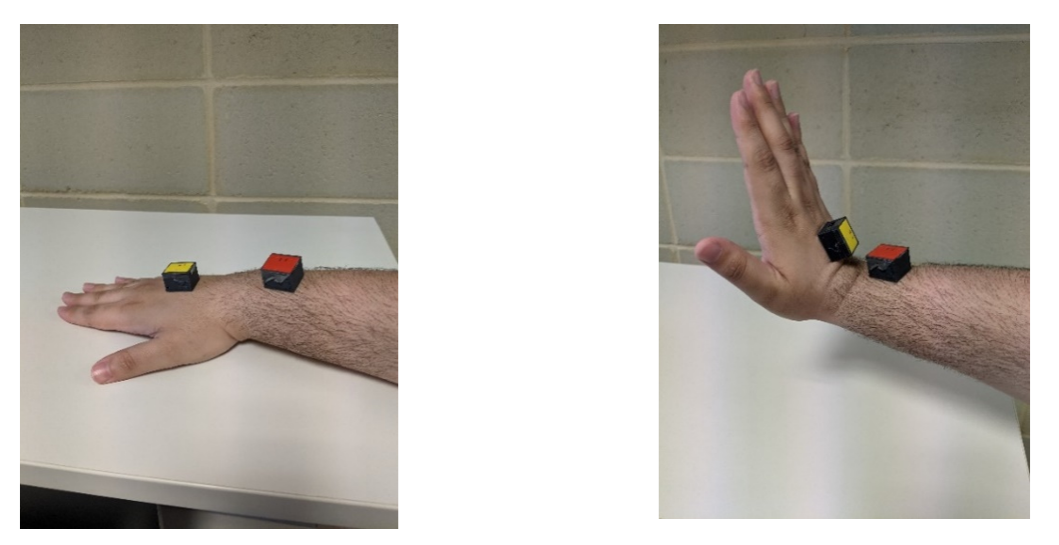
Stop sign motion required by the participants.

Figures 9 and 10 show the raw data captured for a stop sign motion trial of a participant without CP, starting from the stationary position to a stop sign and again to a stationary position. These data included the accelerometer and gyroscope in 3 axes. Figure 11 shows the placement of the sensors on the hand and above the wrist.

**Figure 12:**
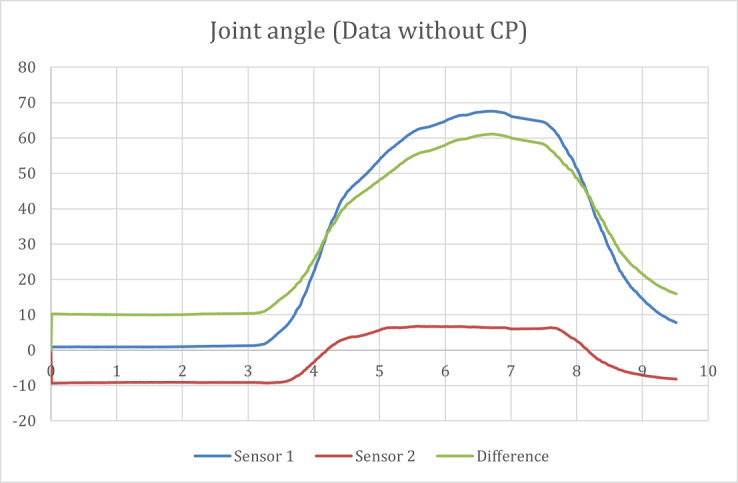
Joint angle results from a participant without CP. CP: cerebral palsy.

After the data were captured, they were processed and run through the different equations described in the joint calculation section of the report. Through these calculations, the drift was removed, and the joint angle was calculated, the results of which are shown in Figure 12.

**Figure 13:**
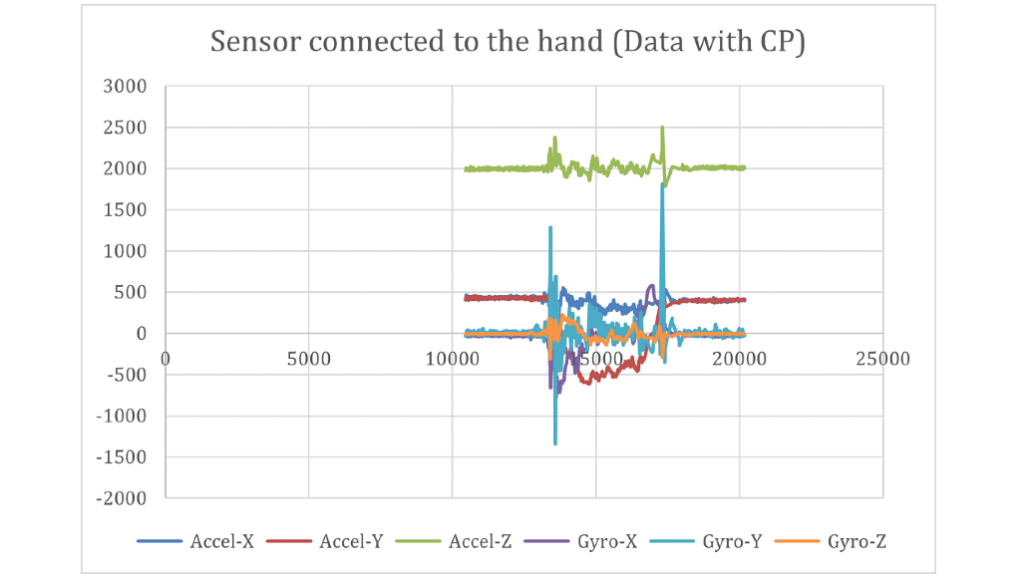
Raw data captured with the sensor connected to the hand (data with CP). CP: cerebral palsy.

**Figure 14:**
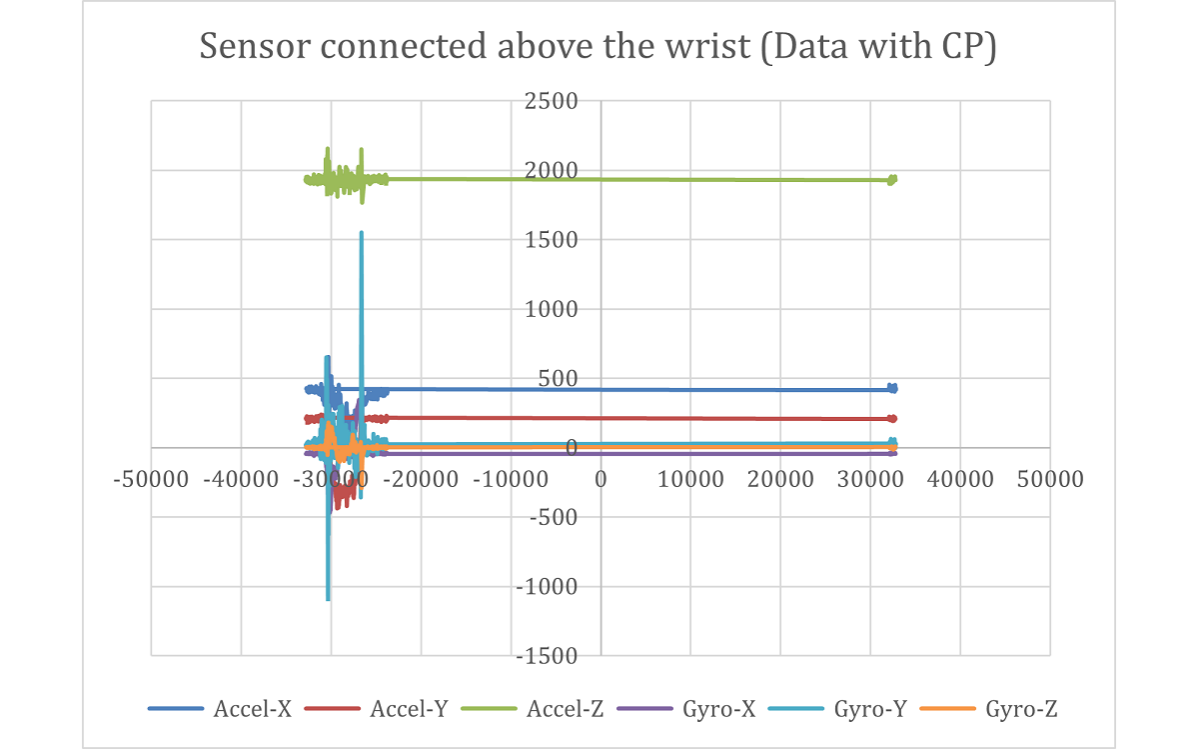
Raw data captured with the sensor connected above the wrist (data with CP). CP: cerebral palsy.

**Figure 15:**
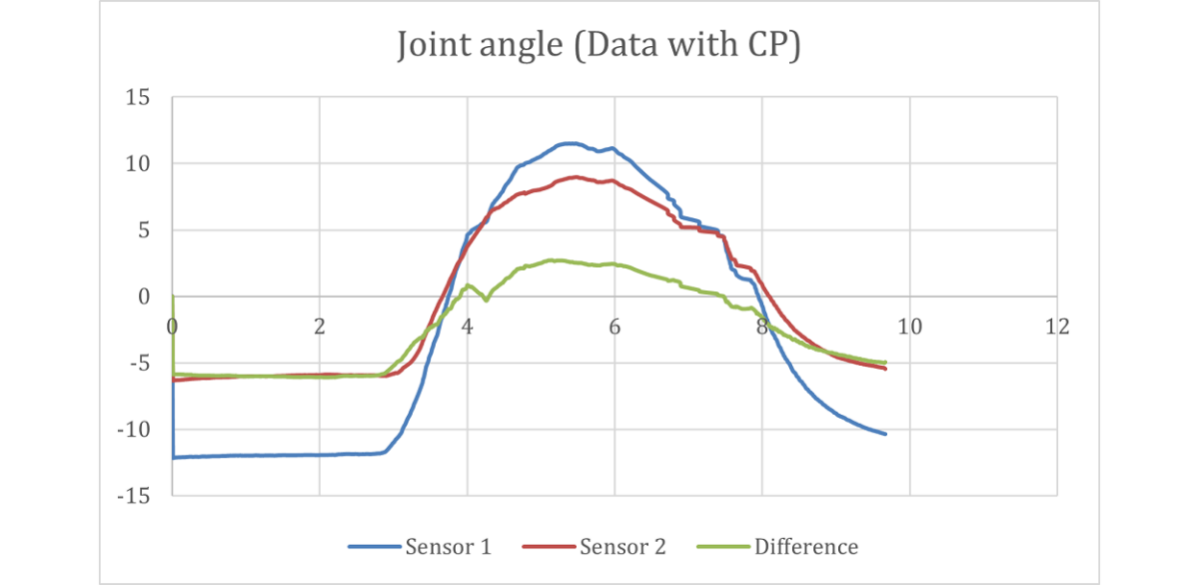
Joint angle results from a participant with CP. CP: cerebral palsy.

The stop sign trials from participants with CP were captured using the same IMUs as those used in the previous group. The results of the raw data captured from the CP participants are shown in Figures 13 and 14. The results of the calculated joint angles are shown in Figure 15.

Anecdotal feedback from MIT and iWHOT researchers was positive about the potential of IMUs to contribute accurate data about active ROM, especially in children for whom goniometric methods are challenging.

After the initial angles were calculated, several classical ML models were trained to create a classifier for the captured data. The Waikato Environment for Knowledge Analysis platform [34] version 3.8 was chosen as the platform for these experiments. Waikato Environment for Knowledge Analysis is a collection of open-source ML algorithms and contains tools for data preparation, classification, regression, clustering, association rule mining, and visualization [34]. The algorithms used consisted of ZeroR, OneR, Bayes Net, Naïve Bays, logistic regression, C4.5 decision tree, random forest, support vector machine, multilayer perceptron, and k-nearest neighbors. The authors analysis of the produced ML results can be found in the *Discussion* section of this paper.

## Discussion

### Principal Findings

The resultant evaluation metrics are accuracy, the number of correctly classified instances over the total number of instances, the area under the curve (AUC), and the area under the receiver operating characteristic (ROC) curve. The ROC curve maps the true positive rates as the x-coordinate and false positive rates as the y-coordinate. Ten-fold cross-validation was adopted, splitting the data set into 10 parts, training the models with 9 parts, and testing with 1 part each time for a total of 10 times. The accuracy and AUC were obtained by averaging the 10 sets of results and taking the weighted average of the 2 classes. The baseline of the experiments was obtained from ZeroR, a classifier that predicts the class that occurs most often in the training data set as the label without considering other features.

**Figure 16:**
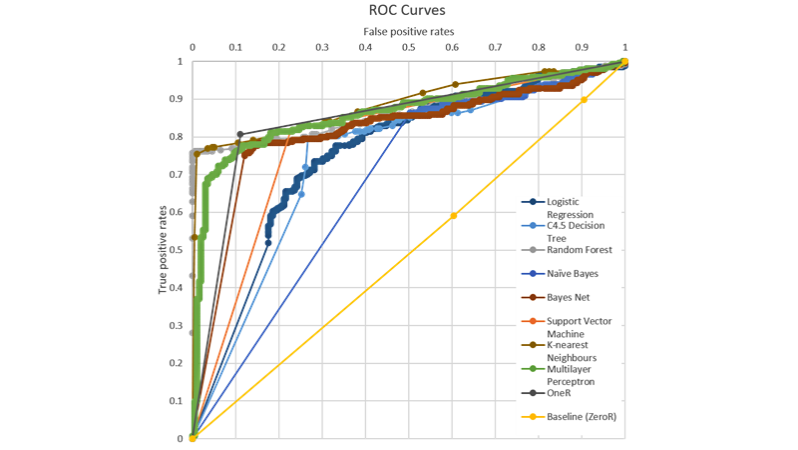
The ROC curves of 10 classification algorithms using the Minimising Impairment Trial data. The area under the curve values are the areas between the ROC curves and the x-axis. ROC: receiver operating characteristic.

Table 3 presents the results of the 9-ML algorithms on the classification using the MIT data. The baseline obtained from ZeroR showed 57.02% accuracy and 0.493 AUC. The best accuracy was 85.75% yielded by random forest, and the best AUC was 0.890 yielded by k-nearest neighbors. Figure 16 shows the ROC curves of the 9 ML algorithms and the baseline. OneR, k-nearest neighbors, multilayer perception, and random forest all produce reasonable ROC curves and are expected to perform well for the problem. Naïve Bayes performs better than the other algorithms owing to the conditional independence assumption it makes. Because the frequency space features are interrelated, it is unreasonable to make this assumption.

**Table 3:** Machine learning result using minimizing impairment training data, showing the best accuracy.

Algorithm	Accuracy (%)	AUC^a^
OneR	84.23	0.848
Logistic regression	72.79	0.749
Naïve Bayes	65.23	0.752
Bayes Net	80.99	0.832
C4.5 decision tree	74.95	0.740
*Random forest* ^b^	*85.75*	0.867
Multilayer perceptron	80.35	0.865
Support vector machine	79.70	0.794
K-nearest neighbors	82.07	*0.890*
*Average*	*78.45*	*0.815*

^a^AUC: area under the curve.

^b^The best accuracy and area under the curve values are italicized.

Curiously, OneR uses only a single feature and achieves 84.23% classification accuracy. The algorithm uses the 91st feature, which is the phase shift corresponding to the second harmonic obtained from the hand sensor. This phenomenon may indicate that the most useful information for classification is recorded by the hand sensor and that omitting one sensor may be possible in the future.

**Figure 17:**
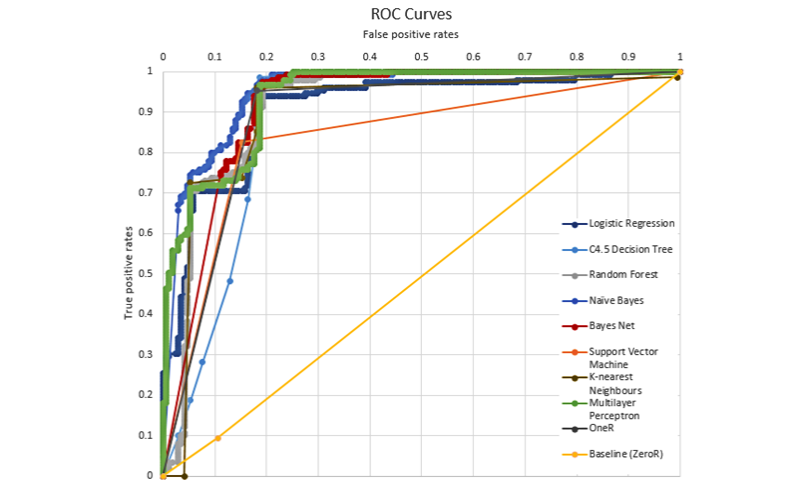
The ROC curves of 10 classification algorithms using the Infant Wrist Hand Orthosis Trial data. The area under the curve values are the areas between the ROC curves and the x-axis. ROC: receiver operating characteristic.

Table 4 presents the results of the 9-ML algorithms in binary classification using the iWHOT data. The baseline obtained from ZeroR showed 53.44% accuracy and 0.494 AUC. The best accuracy was obtained by the C4.5 decision tree at 85.75%, and the best AUC was obtained by Naive Bayes at 0.890. Figure 17 shows the ROC curves of the 9-ML algorithms plus the baseline. Although all models appear to be reasonable classifiers for the problem, it is worth noting that OneR, which classifies based on one feature alone, already achieves 88.13% accuracy and 0.886 AUC. The deciding feature is the amplitude of the acceleration in the row direction on the hand sensor, which corresponds to the most important piece of information in a real-world scenario. The relative underperformance of the more sophisticated algorithms, in contrast, may be due to the observed noises in the training data that lead to biases in the learned models. Such noises include the sensors falling off the participant, the participant not following instructions, etc.

**Table 4:** Machine learning result using Infant Wrist Hand Orthosis Trial data.

Algorithm	Accuracy (%)	AUC^a^
OneR	88.13	0.886
Logistic regression	80.94	0.906
Naive Bayes	86.88	*0.943* ^b^
Bayes Net	88.43	0.921
*C4.5 decision tree*	*89.38*	0.858
Random forest	81.88	0.917
Multilayer perceptron	81.25	0.937
Support vector machine	83.75	0.783
K-nearest neighbors	83.44	0.896
*Average*	*84.90*	*0.894*

^a^AUC: area under the curve.

^b^The best accuracy and area under the curve values are italicized.

### Conclusions

Upon completion of the project, the wrist joint angle was successfully calculated, and CP movement was classified as a feature using ML on raw IMU data**.** Anecdotal positive feedback from MIT and iWHOT researchers was also received regarding the potential for IMUs to contribute accurate data about active ROM, especially where the use of goniometers can be challenging. There may also be the potential to use IMUs for continued monitoring of hand movements throughout the day. The sensor size needs to be reduced to make it more comfortable to wear. Examples of ML and IMU data captured for medical purposes can be seen in the paper titled *Classification of foot drop gait characteristic due to lumbar radiculopathy using machine learning algorithms* [23]. This paper looks at the classification of IMU data captured from hospital patients with foot drop issues using supervised learning and uses 11 different ML classifiers and shows that random forest was the most accurate method with an accuracy of 88.45% for a specific data set [23]. Some of the other ML algorithms used were SVM, Naive Bayers, and deep learning, which gave accuracies of 86.87%, 86.87%, and 86.06%, respectively [14]. Bidabadi et al [30] showed results were very similar to the current findings, although the focus was on a different joint. This suggests that decision-tree-based ML algorithms may be the best option for classifying IMU data for joint movement. The classifier used in this study would be able to distinguish atypical and reduced movement, which can potentially be useful for people with different joint movement disorders such as arthritis and Parkinson disease.

There are some limitations to the IMU setup used in this study, such as the inherent drift of IMUs, which can be corrected by the drift mitigation techniques described in the methods. These techniques may prove problematic for longer trials. There were other issues during the data collection sessions, such as touching the 2 (hand and forearm) sensors because of the small hands of some participants or accidental touching of the sensors by the therapist while using the goniometer, which leads to an increase in noise in the data. Bugs in the data collection interface created for technicians also resulted in some corrupted data and data loss, which added to the preprocessing time of the ML section of this study. Finally, at the initial stages of the project, the scale of the accelerometer was set at +2 g because the slower moving trials rarely reached this value. Once free play situations were introduced that would usually contain rapid movement, particularly in younger children, it was observed that the scale of g needed to be extended beyond this threshold, which resulted in reduced accuracy. This reduction caused some data loss, so the scale was switched to +16 g for faster trials.

As part of future work, real-time calculation of joint angle and orientation data can be implemented so that direct quaternions can be collected and used for this calculation. The research team involved in this paper began the preliminary work on this next step and plans to publish their results once the solution has been fully created. The sensor setup will also be updated to remove the reliance on a separate receiver dongle by switching the communication module to Bluetooth Low Energy transfer to a smartphone application. These changes to the user experience and the medium of transfer would improve the utility of the process of data collection, better continued monitoring of children with CP, and quicker trial sessions in routine appointments for children with CP.

## Appendix

Multimedia Appendix 1 CONSORT-eHEALTH checklist (V 1.6.1).

## Abbreviations

AUC: area under the curve
CP: cerebral palsy
I2C: interintegrated circuit
IMU: inertial measurement unit
IR: infrared
iWHOT: Infant Wrist Hand Orthosis Trial
MIT: Minimising Impairment Trial
ML: machine learning
RCT: randomized controlled trial
RF: radio frequency
ROC: receiver operating characteristic
ROM: range of motion
SPI: serial peripheral interface
Weka: Waikato Environment for Knowledge Analysis
